# Bis(2-carboxybenzo­ato-κ*O* ^1^)bis­[1-cyclo­propyl-6-fluoro-4-oxo-7-(piperazin-4-ium-1-yl)-1,4-dihydro­quinoline-3-carboxyl­ato-κ^2^ *O* ^3^,*O* ^4^]manganese(II) dihydrate

**DOI:** 10.1107/S1600536811011615

**Published:** 2011-04-07

**Authors:** Guang-Ju Zhang, Jiang-Hong He, Shi-Wei Yan, Dian-Zhen Sun, Hai-Yan Chen

**Affiliations:** a College of Chemistry and Chemical Engineering, Southwest University, Chongqing, 400715, People’s Republic of China

## Abstract

The title compound, [Mn(C_17_H_18_FN_3_O_3_)_2_(C_8_H_5_O_4_)_2_]·2H_2_O or [Mn(cfH)_2_(1,2-Hbdc)_2_]·2H_2_O (cfH = ciprofloxacin = 1-cyclo­propyl-6-fluoro-1,4-dihydro-4-oxo-7-(1-piperazin­yl)-3-quinoline carb­oxy­lic acid, 1,2-bdc = benzene-1,2-dicarboxyl­ate), has been prepared under hydro­thermal conditions. The Mn^2+^ atom, located on an inversion centre, exhibits a distorted octa­hedral geometry, coordinated by four O atoms from two symmetry-related zwitterionic ciprofloxacin ligands in the equatorial positions and two O atoms of two 1,2-Hbdc ligands in the axial positions. The complex mol­ecules are linked into a two-dimensional network through N—H⋯O and OW—H⋯O hydrogen bonds. A strong intramolecular hydrogen bond between the carboxyl/carboxylate groups of the 1,2-Hbdc anion is also present. The layers are further extended through off-set aromatic π–π stacking inter­actions of cfH groups [centroid–centroid distance of 3.657 (2) Å] into the final three-dimensional supra­molecular arrays.

## Related literature

For background to the anti­biotic drug ciprofloxacin, see: Turel (2002 ▶); Xiao *et al.* (2005 ▶). The mechanisms of action of the quinolone anti­bacterial agents are either their inhibition of DNA gyrase (Topoisomerase II) or their inter­action with the DNA mol­ecule *via* a metal complex inter­mediate, see: Chulvi *et al.* (1991 ▶); Ruiz *et al.* (1993 ▶); Wallis *et al.* (1995 ▶). For related structures, see: Fabbiani & Dittrich (2008 ▶); Wang *et al.* (2009 ▶). 
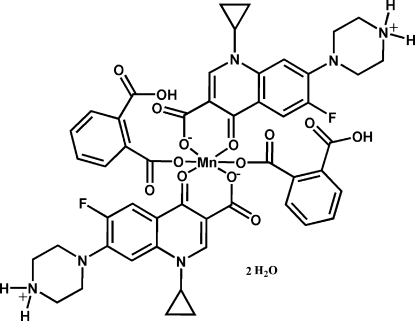


## Experimental

### 

#### Crystal data

[Mn(C_17_H_18_FN_3_O_3_)_2_(C_8_H_5_O_4_)_2_]·2H_2_O
*M* *_r_* = 1083.90
Monoclinic, 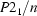

*a* = 9.4510 (19) Å
*b* = 22.042 (4) Å
*c* = 11.695 (2) Å
β = 98.44 (3)°
*V* = 2409.9 (8) Å^3^
*Z* = 2
Mo *K*α radiation
μ = 0.36 mm^−1^
*T* = 293 K
0.58 × 0.47 × 0.32 mm

#### Data collection

Rigaku R-AXIS RAPID IP diffractometer
Absorption correction: multi-scan (*SADABS*; Sheldrick, 1996 ▶) *T*_min_ = 0.817, *T*_max_ = 0.893
22913 measured reflections
5494 independent reflections
3555 reflections with *I* > 2σ(*I*)
*R*_int_ = 0.068

#### Refinement

*R*[*F* ^2^ > 2σ(*F* ^2^)] = 0.051
*wR*(*F* ^2^) = 0.145
*S* = 1.00
5494 reflections
350 parameters
10 restraints
H atoms treated by a mixture of independent and constrained refinement
Δρ_max_ = 0.45 e Å^−3^
Δρ_min_ = −0.36 e Å^−3^

### 

Data collection: *RAPID-AUTO* (Rigaku, 1998 ▶); cell refinement: *RAPID-AUTO*; data reduction: *RAPID-AUTO*; program(s) used to solve structure: *SHELXS97* (Sheldrick, 2008 ▶); program(s) used to refine structure: *SHELXL97* (Sheldrick, 2008 ▶); molecular graphics: *SHELXTL* (Sheldrick, 2008 ▶); software used to prepare material for publication: *SHELXTL*.

## Supplementary Material

Crystal structure: contains datablocks I, global. DOI: 10.1107/S1600536811011615/vm2075sup1.cif

Structure factors: contains datablocks I. DOI: 10.1107/S1600536811011615/vm2075Isup2.hkl

Additional supplementary materials:  crystallographic information; 3D view; checkCIF report

## Figures and Tables

**Table 1:** Hydrogen-bond geometry (Å, °)

*D*—H⋯*A*	*D*—H	H⋯*A*	*D*⋯*A*	*D*—H⋯*A*
O*W*1—H*W*1*B*⋯O5	0.84 (3)	2.43 (3)	3.108 (4)	139 (4)
N1—H1*A*⋯O7^i^	0.90	1.82	2.717 (3)	174
N1—H1*B*⋯O1^ii^	0.90	1.79	2.688 (3)	173
N1—H1*B*⋯O2^ii^	0.90	2.60	3.246 (3)	130
O*W*1—H*W*1*A*⋯O3^iii^	0.84 (3)	2.12 (1)	2.937 (3)	164 (4)
O6—H6⋯O5	0.85 (4)	1.53 (4)	2.379 (4)	175 (8)

Symmetry codes: (i) 
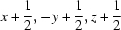
; (ii) 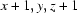; (iii) 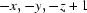.

**Fig. 1.**
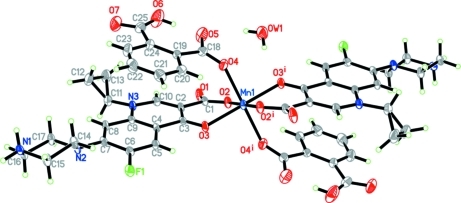
ORTEP drawing of 1 with thermal ellipsoids at 30% probability. Symmetry code (i): -x, -y, -z+1.

**Fig. 2.**
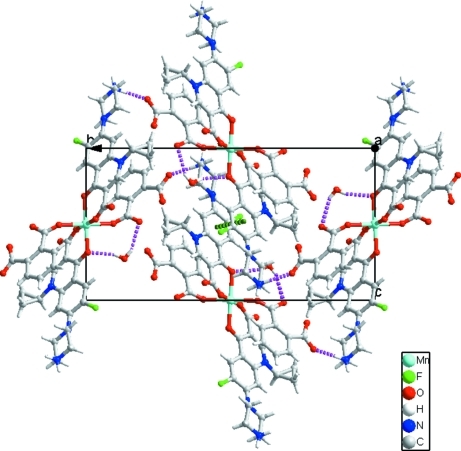
Perspective view of the three-dimensional supramolecular network in 1. The hydrogen bonds are indicated by pink dotted lines while the aromatic π—π stacking interaction is indicated by a green dotted line.

## supplementary crystallographic information

### Comment

Ciprofloxacin [cfH = 1-cyclopropyl-6-fluoro-1,4-dihydro-4-oxo-7-(1-piperazinyl)-3-quinoline carboxylic acid] belongs to the quinolone family of synthetic antibiotics (Turel, 2002; Xiao *et al.*, 2005) and is a third generation quinolone antibacterial drug with broad-spectrum antibacterial activity (especially aerobic gram-negative bacilli high antibacterial activity). The mechanisms of action of the quinolone antibacterial agents are either their inhibition of DNA gyrase (Topoisomerase II), an essential bacterial enzyme that maintains superhelical twists in DNA, or their interaction with the DNA molecule *via* a metal complex intermediate (Chulvi *et al.*, 1991; Ruiz *et al.*, 1993; Wallis *et al.*, 1995).

The Ciprofloxacin and its deprotonated anions can show a number of different coordinating or bridging modes. The title complex consists of a Mn^2+^ atom lying on an inversion center, two Ciprofloxacin ligands, two 1,2-benzenedicarboxylate ligands (1,2-bdc) and a water molecule (Fig. 1). The Ciprofloxacin ligand acts as chelating bidentate and the Mn(II) atom is coordinated by four oxygen atoms from two different Ciprofloxacin ligands and two oxygen atoms from two 1,2-bdc ligands. The N—H···O and OW—H···O hydrogen bonds link the discrete molecules into two-dimensional arrays (Table 1). These two-dimensional layers are further extended through off-set aromatic π—π stacking interplanar of cfH groups (centroid distance of 3.657 Å) into the final three-dimensional supramolecular arrays (Fig. 2).

### Experimental

A mixture of Mn(OAc)_2_.4H_2_O (0.5 mmol), ciprofloxacin hydrochloride (0.25 mmol), 1,2-KHbdc (0.5 mmol), and water (7 ml) was stirred for 30 min in air (solution pH = 4.0), then transferred and sealed in an 18 ml Teflon-lined autoclave, which was heated at 110 °C for 96 h. After slow cooling to room temperature, yellow block crystals were filtered off, washed with distilled water, and dried at ambient temperature.

### Refinement

The structure was solved by direct methods and successive Fourier difference synthesis. The H atoms bonded to C or N atoms were positioned geometrically and refined using a riding model [aromatic C—H = 0.93 Å, aliphatic C—H = 0.97 – 0.98 Å and N—H = 0.90 Å, *U*_iso_(H) = 1.2U_eq_(C) and *U*_iso_(H) = 1.2U_eq_(N)]. The H atoms bonded to OW atoms were located in a difference Fourier maps and refined with OW—H = 0.84 Å and U_iso_(H) = 1.3U_eq_(OW).

### Crystal data

**Table:**

[Mn(C_17_H_18_FN_3_O_3_)_2_(C_8_H_5_O_4_)_2_]·2H_2_O	*F*(000) = 1126
*M**_r_* = 1083.90	*D*_x_ = 1.494 Mg m^−^^3^
Monoclinic, *P*2_1_/*n*	Mo *K*α radiation, λ = 0.71073 Å
Hall symbol: -P 2yn	Cell parameters from 22913 reflections
*a* = 9.4510 (19) Å	θ = 3.1–27.5°
*b* = 22.042 (4) Å	µ = 0.36 mm^−^^1^
*c* = 11.695 (2) Å	*T* = 293 K
β = 98.44 (3)°	Block, yellow
*V* = 2409.9 (8) Å^3^	0.58 × 0.47 × 0.32 mm
*Z* = 2	

### Data collection

**Table:**

Rigaku R-AXIS RAPID IP diffractometer	5494 independent reflections
Radiation source: fine-focus sealed tube	3555 reflections with *I* > 2σ(*I*)
graphite	*R*_int_ = 0.068
Detector resolution: 100x100 microns pixels mm^-1^	θ_max_ = 27.5°, θ_min_ = 3.1°
Oscillation scans	*h* = −12→12
Absorption correction: multi-scan (*SADABS*; Sheldrick, 1996)	*k* = −28→27
*T*_min_ = 0.817, *T*_max_ = 0.893	*l* = −14→15
22913 measured reflections	

### Refinement

**Table:**

Refinement on *F*^2^	Primary atom site location: structure-invariant direct methods
Least-squares matrix: full	Secondary atom site location: difference Fourier map
*R*[*F*^2^ > 2σ(*F*^2^)] = 0.051	Hydrogen site location: inferred from neighbouring sites
*wR*(*F*^2^) = 0.145	H atoms treated by a mixture of independent and constrained refinement
*S* = 1.00	*w* = 1/[σ^2^(*F*_o_^2^) + (0.080*P*)^2^] where *P* = (*F*_o_^2^ + 2*F*_c_^2^)/3
5494 reflections	(Δ/σ)_max_ < 0.001
350 parameters	Δρ_max_ = 0.45 e Å^−^^3^
10 restraints	Δρ_min_ = −0.36 e Å^−^^3^

### Special details

**Table:**

Geometry. All esds (except the esd in the dihedral angle between two l.s. planes) are estimated using the full covariance matrix. The cell esds are taken into account individually in the estimation of esds in distances, angles and torsion angles; correlations between esds in cell parameters are only used when they are defined by crystal symmetry. An approximate (isotropic) treatment of cell esds is used for estimating esds involving l.s. planes.
Refinement. Refinement of F^2^ against ALL reflections. The weighted R-factor wR and goodness of fit S are based on F^2^, conventional R-factors R are based on F, with F set to zero for negative F^2^. The threshold expression of F^2^ > 2sigma(F^2^) is used only for calculating R-factors(gt) etc. and is not relevant to the choice of reflections for refinement. R-factors based on F^2^ are statistically about twice as large as those based on F, and R- factors based on ALL data will be even larger.

### Fractional atomic coordinates and isotropic or equivalent isotropic displacement parameters (Å^2^)

**Table:**

	*x*	*y*	*z*	*U*_iso_*/*U*_eq_	
Mn1	0.0000	0.0000	0.5000	0.02941 (16)	
F1	0.36711 (17)	−0.03305 (7)	1.05085 (13)	0.0405 (4)	
O1	−0.3533 (2)	0.08833 (10)	0.61415 (16)	0.0463 (5)	
OW1	−0.1255 (4)	0.13296 (13)	0.2728 (2)	0.0820 (9)	
HW1A	−0.094 (5)	0.0991 (10)	0.298 (3)	0.110*	
HW1B	−0.138 (5)	0.1555 (15)	0.328 (2)	0.109*	
O2	−0.20086 (19)	0.03315 (9)	0.53302 (15)	0.0357 (4)	
O3	0.0395 (2)	−0.00587 (8)	0.68591 (15)	0.0343 (4)	
O4	0.0782 (2)	0.09397 (9)	0.50461 (18)	0.0439 (5)	
O5	−0.0132 (3)	0.18400 (11)	0.5170 (2)	0.0750 (8)	
O6	−0.0126 (4)	0.26486 (14)	0.6518 (3)	0.1005 (12)	
O7	0.0876 (4)	0.28742 (12)	0.8236 (3)	0.0931 (11)	
N1	0.5198 (2)	0.10018 (9)	1.39405 (17)	0.0314 (5)	
H1A	0.5482	0.1366	1.3711	0.038*	
H1B	0.5652	0.0931	1.4660	0.038*	
N2	0.3244 (2)	0.06259 (10)	1.19684 (17)	0.0305 (5)	
N3	−0.0836 (2)	0.12018 (9)	0.91028 (17)	0.0298 (5)	
C1	−0.2347 (3)	0.06311 (11)	0.6166 (2)	0.0295 (5)	
C2	−0.1306 (3)	0.06868 (11)	0.7272 (2)	0.0276 (5)	
C3	−0.0053 (3)	0.03176 (11)	0.75371 (19)	0.0259 (5)	
C4	0.0734 (2)	0.03979 (11)	0.8693 (2)	0.0259 (5)	
C5	0.1887 (3)	0.00113 (11)	0.9079 (2)	0.0286 (5)	
H5A	0.2133	−0.0297	0.8601	0.034*	
C6	0.2637 (3)	0.00873 (11)	1.0142 (2)	0.0285 (5)	
C7	0.2384 (3)	0.05589 (11)	1.0898 (2)	0.0277 (5)	
C8	0.1208 (3)	0.09297 (11)	1.0534 (2)	0.0298 (5)	
H8A	0.0973	0.1239	1.1015	0.036*	
C9	0.0374 (3)	0.08415 (11)	0.9447 (2)	0.0259 (5)	
C10	−0.1624 (3)	0.10961 (12)	0.8066 (2)	0.0315 (5)	
H10A	−0.2456	0.1322	0.7878	0.038*	
C11	−0.1357 (3)	0.16131 (13)	0.9927 (2)	0.0404 (6)	
H11A	−0.1788	0.1415	1.0542	0.048*	
C12	−0.0615 (4)	0.21907 (14)	1.0259 (3)	0.0578 (9)	
H12A	0.0223	0.2289	0.9906	0.069*	
H12B	−0.0577	0.2326	1.1052	0.069*	
C13	−0.2032 (4)	0.21968 (15)	0.9507 (3)	0.0606 (10)	
H13A	−0.2854	0.2334	0.9844	0.073*	
H13B	−0.2054	0.2297	0.8697	0.073*	
C14	0.4800 (3)	0.06480 (13)	1.1950 (2)	0.0358 (6)	
H14A	0.5066	0.1044	1.1691	0.043*	
H14B	0.5065	0.0346	1.1416	0.043*	
C15	0.5581 (3)	0.05253 (13)	1.3150 (2)	0.0384 (6)	
H15A	0.5314	0.0130	1.3412	0.046*	
H15B	0.6606	0.0527	1.3140	0.046*	
C16	0.3629 (3)	0.10097 (13)	1.3950 (2)	0.0346 (6)	
H16A	0.3392	0.1339	1.4439	0.041*	
H16B	0.3341	0.0632	1.4276	0.041*	
C17	0.2812 (3)	0.10894 (13)	1.2747 (2)	0.0349 (6)	
H17A	0.1793	0.1057	1.2772	0.042*	
H17B	0.2999	0.1489	1.2457	0.042*	
C18	0.0744 (3)	0.14291 (12)	0.5553 (2)	0.0376 (6)	
C19	0.1798 (3)	0.15346 (11)	0.6640 (2)	0.0321 (6)	
C20	0.2873 (3)	0.10976 (13)	0.6846 (3)	0.0399 (6)	
H20A	0.2873	0.0778	0.6327	0.048*	
C21	0.3928 (4)	0.11212 (16)	0.7781 (3)	0.0531 (8)	
H21A	0.4638	0.0826	0.7883	0.064*	
C22	0.3934 (4)	0.15842 (18)	0.8571 (3)	0.0626 (10)	
H22A	0.4640	0.1601	0.9214	0.075*	
C23	0.2886 (4)	0.20213 (16)	0.8400 (3)	0.0573 (9)	
H23A	0.2890	0.2330	0.8943	0.069*	
C24	0.1812 (3)	0.20172 (12)	0.7437 (2)	0.0394 (6)	
C25	0.0795 (4)	0.25462 (14)	0.7399 (3)	0.0562 (9)	
H6	−0.017 (6)	0.2366 (18)	0.602 (4)	0.13 (2)*	

### Atomic displacement parameters (Å^2^)

**Table:**

	*U*^11^	*U*^22^	*U*^33^	*U*^12^	*U*^13^	*U*^23^
Mn1	0.0300 (3)	0.0375 (3)	0.0188 (3)	0.0027 (2)	−0.0029 (2)	−0.0051 (2)
F1	0.0374 (8)	0.0479 (9)	0.0314 (8)	0.0147 (7)	−0.0111 (7)	−0.0025 (7)
O1	0.0317 (10)	0.0791 (15)	0.0242 (10)	0.0190 (10)	−0.0087 (8)	−0.0106 (9)
OW1	0.122 (3)	0.0689 (17)	0.0486 (16)	0.0195 (17)	−0.0109 (16)	0.0000 (13)
O2	0.0290 (9)	0.0515 (11)	0.0236 (9)	0.0050 (8)	−0.0064 (7)	−0.0084 (8)
O3	0.0407 (10)	0.0397 (10)	0.0193 (9)	0.0100 (8)	−0.0059 (7)	−0.0051 (7)
O4	0.0489 (12)	0.0417 (11)	0.0405 (12)	−0.0045 (9)	0.0045 (9)	−0.0119 (8)
O5	0.0855 (19)	0.0603 (15)	0.0650 (17)	0.0255 (14)	−0.0361 (14)	−0.0108 (12)
O6	0.115 (3)	0.0683 (19)	0.106 (3)	0.0507 (19)	−0.027 (2)	−0.0308 (18)
O7	0.152 (3)	0.0554 (15)	0.073 (2)	0.0280 (18)	0.021 (2)	−0.0228 (14)
N1	0.0332 (11)	0.0370 (11)	0.0207 (10)	−0.0053 (9)	−0.0077 (8)	0.0009 (8)
N2	0.0263 (10)	0.0451 (12)	0.0178 (10)	0.0021 (9)	−0.0045 (8)	−0.0056 (8)
N3	0.0305 (11)	0.0366 (11)	0.0204 (10)	0.0057 (9)	−0.0021 (8)	−0.0045 (8)
C1	0.0260 (12)	0.0396 (13)	0.0200 (12)	0.0012 (10)	−0.0056 (10)	−0.0004 (9)
C2	0.0255 (12)	0.0378 (13)	0.0177 (11)	0.0011 (10)	−0.0032 (9)	−0.0009 (9)
C3	0.0263 (12)	0.0325 (12)	0.0171 (11)	−0.0031 (10)	−0.0023 (9)	0.0015 (9)
C4	0.0241 (11)	0.0351 (12)	0.0171 (11)	−0.0007 (10)	−0.0019 (9)	0.0005 (9)
C5	0.0282 (12)	0.0336 (12)	0.0226 (12)	0.0043 (11)	−0.0015 (9)	−0.0034 (10)
C6	0.0242 (11)	0.0358 (13)	0.0242 (12)	0.0051 (10)	−0.0011 (9)	0.0029 (9)
C7	0.0247 (12)	0.0385 (13)	0.0176 (11)	−0.0020 (10)	−0.0041 (9)	0.0016 (9)
C8	0.0295 (12)	0.0390 (13)	0.0192 (12)	0.0026 (11)	−0.0019 (10)	−0.0051 (9)
C9	0.0254 (11)	0.0316 (12)	0.0193 (11)	0.0011 (10)	−0.0018 (9)	0.0000 (9)
C10	0.0268 (12)	0.0401 (13)	0.0253 (13)	0.0049 (11)	−0.0040 (10)	0.0004 (10)
C11	0.0410 (15)	0.0462 (16)	0.0326 (15)	0.0093 (13)	0.0010 (12)	−0.0077 (12)
C12	0.068 (2)	0.0489 (18)	0.052 (2)	0.0073 (17)	−0.0037 (17)	−0.0163 (15)
C13	0.073 (2)	0.0552 (19)	0.050 (2)	0.0284 (18)	−0.0030 (18)	−0.0135 (15)
C14	0.0240 (12)	0.0563 (17)	0.0253 (13)	−0.0002 (11)	−0.0023 (10)	−0.0054 (11)
C15	0.0285 (13)	0.0539 (17)	0.0298 (14)	0.0020 (12)	−0.0053 (11)	−0.0067 (12)
C16	0.0343 (14)	0.0467 (15)	0.0211 (13)	−0.0018 (12)	−0.0011 (11)	−0.0045 (10)
C17	0.0279 (12)	0.0505 (15)	0.0235 (13)	0.0024 (11)	−0.0050 (10)	−0.0080 (11)
C18	0.0369 (14)	0.0368 (14)	0.0389 (16)	−0.0007 (12)	0.0046 (12)	−0.0013 (11)
C19	0.0358 (14)	0.0326 (13)	0.0288 (13)	−0.0023 (11)	0.0075 (11)	−0.0012 (10)
C20	0.0406 (15)	0.0419 (15)	0.0373 (16)	0.0048 (12)	0.0062 (12)	0.0008 (12)
C21	0.0460 (18)	0.063 (2)	0.0484 (19)	0.0104 (16)	0.0003 (15)	0.0069 (15)
C22	0.056 (2)	0.083 (3)	0.0423 (19)	−0.0044 (19)	−0.0149 (16)	−0.0008 (17)
C23	0.066 (2)	0.058 (2)	0.046 (2)	−0.0099 (17)	0.0002 (17)	−0.0156 (15)
C24	0.0456 (16)	0.0369 (14)	0.0355 (15)	−0.0026 (13)	0.0052 (13)	−0.0046 (11)
C25	0.078 (3)	0.0341 (16)	0.058 (2)	0.0072 (16)	0.0164 (19)	−0.0045 (14)

### Geometric parameters (Å, °)

**Table:**

Mn1—O2	2.1219 (18)	C6—C7	1.408 (3)
Mn1—O2^i^	2.1219 (18)	C7—C8	1.394 (3)
Mn1—O3	2.1552 (17)	C8—C9	1.408 (3)
Mn1—O3^i^	2.1552 (17)	C8—H8A	0.9300
Mn1—O4	2.1971 (19)	C10—H10A	0.9300
Mn1—O4^i^	2.1971 (19)	C11—C12	1.478 (4)
F1—C6	1.365 (3)	C11—C13	1.488 (4)
O1—C1	1.247 (3)	C11—H11A	0.9800
OW1—HW1A	0.84 (3)	C12—C13	1.491 (5)
OW1—HW1B	0.84 (3)	C12—H12A	0.9700
O2—C1	1.259 (3)	C12—H12B	0.9700
O3—C3	1.263 (3)	C13—H13A	0.9700
O4—C18	1.234 (3)	C13—H13B	0.9700
O5—C18	1.264 (4)	C14—C15	1.510 (4)
O6—C25	1.268 (5)	C14—H14A	0.9700
O6—H6	0.85 (4)	C14—H14B	0.9700
O7—C25	1.210 (4)	C15—H15A	0.9700
N1—C15	1.480 (3)	C15—H15B	0.9700
N1—C16	1.485 (3)	C16—C17	1.513 (3)
N1—H1A	0.9000	C16—H16A	0.9700
N1—H1B	0.9000	C16—H16B	0.9700
N2—C7	1.396 (3)	C17—H17A	0.9700
N2—C17	1.466 (3)	C17—H17B	0.9700
N2—C14	1.475 (3)	C18—C19	1.514 (4)
N3—C10	1.346 (3)	C19—C20	1.395 (4)
N3—C9	1.402 (3)	C19—C24	1.413 (4)
N3—C11	1.460 (3)	C20—C21	1.369 (4)
C1—C2	1.511 (3)	C20—H20A	0.9300
C2—C10	1.360 (3)	C21—C22	1.376 (5)
C2—C3	1.433 (3)	C21—H21A	0.9300
C3—C4	1.455 (3)	C22—C23	1.375 (5)
C4—C9	1.392 (3)	C22—H22A	0.9300
C4—C5	1.405 (3)	C23—C24	1.402 (4)
C5—C6	1.349 (3)	C23—H23A	0.9300
C5—H5A	0.9300	C24—C25	1.508 (4)
			
O2—Mn1—O2^i^	180.00 (9)	C12—C11—C13	60.4 (2)
O2—Mn1—O3	82.19 (7)	N3—C11—H11A	115.1
O2^i^—Mn1—O3	97.81 (7)	C12—C11—H11A	115.1
O2—Mn1—O3^i^	97.81 (7)	C13—C11—H11A	115.1
O2^i^—Mn1—O3^i^	82.19 (7)	C11—C12—C13	60.2 (2)
O3—Mn1—O3^i^	180.0	C11—C12—H12A	117.8
O2—Mn1—O4	88.72 (8)	C13—C12—H12A	117.8
O2^i^—Mn1—O4	91.28 (8)	C11—C12—H12B	117.8
O3—Mn1—O4	91.37 (7)	C13—C12—H12B	117.8
O3^i^—Mn1—O4	88.63 (7)	H12A—C12—H12B	114.9
O2—Mn1—O4^i^	91.28 (8)	C11—C13—C12	59.5 (2)
O2^i^—Mn1—O4^i^	88.72 (8)	C11—C13—H13A	117.8
O3—Mn1—O4^i^	88.63 (7)	C12—C13—H13A	117.8
O3^i^—Mn1—O4^i^	91.37 (7)	C11—C13—H13B	117.8
O4—Mn1—O4^i^	180.0	C12—C13—H13B	117.8
HW1A—OW1—HW1B	109.7 (18)	H13A—C13—H13B	115.0
C1—O2—Mn1	131.15 (16)	N2—C14—C15	109.5 (2)
C3—O3—Mn1	124.73 (15)	N2—C14—H14A	109.8
C18—O4—Mn1	143.4 (2)	C15—C14—H14A	109.8
C25—O6—H6	113 (4)	N2—C14—H14B	109.8
C15—N1—C16	110.5 (2)	C15—C14—H14B	109.8
C15—N1—H1A	109.6	H14A—C14—H14B	108.2
C16—N1—H1A	109.6	N1—C15—C14	108.8 (2)
C15—N1—H1B	109.6	N1—C15—H15A	109.9
C16—N1—H1B	109.6	C14—C15—H15A	109.9
H1A—N1—H1B	108.1	N1—C15—H15B	109.9
C7—N2—C17	116.8 (2)	C14—C15—H15B	109.9
C7—N2—C14	116.2 (2)	H15A—C15—H15B	108.3
C17—N2—C14	110.8 (2)	N1—C16—C17	111.6 (2)
C10—N3—C9	118.7 (2)	N1—C16—H16A	109.3
C10—N3—C11	120.4 (2)	C17—C16—H16A	109.3
C9—N3—C11	120.1 (2)	N1—C16—H16B	109.3
O1—C1—O2	123.1 (2)	C17—C16—H16B	109.3
O1—C1—C2	116.9 (2)	H16A—C16—H16B	108.0
O2—C1—C2	120.0 (2)	N2—C17—C16	110.2 (2)
C10—C2—C3	119.2 (2)	N2—C17—H17A	109.6
C10—C2—C1	117.3 (2)	C16—C17—H17A	109.6
C3—C2—C1	123.5 (2)	N2—C17—H17B	109.6
O3—C3—C2	125.5 (2)	C16—C17—H17B	109.6
O3—C3—C4	119.4 (2)	H17A—C17—H17B	108.1
C2—C3—C4	115.2 (2)	O4—C18—O5	121.3 (3)
C9—C4—C5	118.2 (2)	O4—C18—C19	118.4 (2)
C9—C4—C3	122.3 (2)	O5—C18—C19	120.3 (2)
C5—C4—C3	119.4 (2)	C20—C19—C24	118.1 (3)
C6—C5—C4	119.9 (2)	C20—C19—C18	114.3 (2)
C6—C5—H5A	120.0	C24—C19—C18	127.6 (2)
C4—C5—H5A	120.0	C21—C20—C19	122.5 (3)
C5—C6—F1	117.4 (2)	C21—C20—H20A	118.7
C5—C6—C7	123.8 (2)	C19—C20—H20A	118.7
F1—C6—C7	118.8 (2)	C20—C21—C22	119.7 (3)
C8—C7—N2	123.0 (2)	C20—C21—H21A	120.2
C8—C7—C6	116.3 (2)	C22—C21—H21A	120.2
N2—C7—C6	120.7 (2)	C23—C22—C21	119.4 (3)
C7—C8—C9	120.7 (2)	C23—C22—H22A	120.3
C7—C8—H8A	119.7	C21—C22—H22A	120.3
C9—C8—H8A	119.7	C22—C23—C24	122.2 (3)
C4—C9—N3	118.7 (2)	C22—C23—H23A	118.9
C4—C9—C8	120.8 (2)	C24—C23—H23A	118.9
N3—C9—C8	120.4 (2)	C23—C24—C19	118.0 (3)
N3—C10—C2	125.6 (2)	C23—C24—C25	113.7 (3)
N3—C10—H10A	117.2	C19—C24—C25	128.3 (3)
C2—C10—H10A	117.2	O7—C25—O6	120.4 (4)
N3—C11—C12	121.0 (3)	O7—C25—C24	118.3 (4)
N3—C11—C13	119.2 (3)	O6—C25—C24	121.3 (3)
			
O2^i^—Mn1—O2—C1	−97 (100)	C5—C4—C9—C8	4.6 (4)
O3—Mn1—O2—C1	31.5 (2)	C3—C4—C9—C8	−176.3 (2)
O3^i^—Mn1—O2—C1	−148.5 (2)	C10—N3—C9—C4	1.5 (3)
O4—Mn1—O2—C1	−60.0 (2)	C11—N3—C9—C4	171.6 (2)
O4^i^—Mn1—O2—C1	120.0 (2)	C10—N3—C9—C8	−178.7 (2)
O2—Mn1—O3—C3	−36.48 (19)	C11—N3—C9—C8	−8.6 (4)
O2^i^—Mn1—O3—C3	143.52 (19)	C7—C8—C9—C4	−2.5 (4)
O3^i^—Mn1—O3—C3	−147 (100)	C7—C8—C9—N3	177.7 (2)
O4—Mn1—O3—C3	52.0 (2)	C9—N3—C10—C2	−4.1 (4)
O4^i^—Mn1—O3—C3	−128.0 (2)	C11—N3—C10—C2	−174.2 (3)
O2—Mn1—O4—C18	39.5 (3)	C3—C2—C10—N3	1.5 (4)
O2^i^—Mn1—O4—C18	−140.5 (3)	C1—C2—C10—N3	179.1 (2)
O3—Mn1—O4—C18	−42.7 (3)	C10—N3—C11—C12	−114.2 (3)
O3^i^—Mn1—O4—C18	137.3 (3)	C9—N3—C11—C12	75.8 (3)
O4^i^—Mn1—O4—C18	−161 (100)	C10—N3—C11—C13	−43.1 (4)
Mn1—O2—C1—O1	167.0 (2)	C9—N3—C11—C13	146.9 (3)
Mn1—O2—C1—C2	−14.3 (4)	N3—C11—C12—C13	108.3 (3)
O1—C1—C2—C10	−12.1 (4)	N3—C11—C13—C12	−111.2 (3)
O2—C1—C2—C10	169.1 (2)	C7—N2—C14—C15	−162.5 (2)
O1—C1—C2—C3	165.3 (2)	C17—N2—C14—C15	61.0 (3)
O2—C1—C2—C3	−13.4 (4)	C16—N1—C15—C14	58.7 (3)
Mn1—O3—C3—C2	26.7 (3)	N2—C14—C15—N1	−61.2 (3)
Mn1—O3—C3—C4	−153.29 (17)	C15—N1—C16—C17	−55.8 (3)
C10—C2—C3—O3	−176.6 (2)	C7—N2—C17—C16	167.0 (2)
C1—C2—C3—O3	5.9 (4)	C14—N2—C17—C16	−56.8 (3)
C10—C2—C3—C4	3.4 (3)	N1—C16—C17—N2	54.2 (3)
C1—C2—C3—C4	−174.0 (2)	Mn1—O4—C18—O5	−95.6 (4)
O3—C3—C4—C9	174.2 (2)	Mn1—O4—C18—C19	85.7 (4)
C2—C3—C4—C9	−5.8 (3)	O4—C18—C19—C20	10.4 (4)
O3—C3—C4—C5	−6.8 (3)	O5—C18—C19—C20	−168.3 (3)
C2—C3—C4—C5	173.2 (2)	O4—C18—C19—C24	−170.2 (3)
C9—C4—C5—C6	−1.9 (4)	O5—C18—C19—C24	11.1 (5)
C3—C4—C5—C6	179.0 (2)	C24—C19—C20—C21	−0.1 (4)
C4—C5—C6—F1	174.8 (2)	C18—C19—C20—C21	179.4 (3)
C4—C5—C6—C7	−3.2 (4)	C19—C20—C21—C22	1.2 (5)
C17—N2—C7—C8	3.5 (4)	C20—C21—C22—C23	−0.8 (5)
C14—N2—C7—C8	−130.4 (3)	C21—C22—C23—C24	−0.8 (6)
C17—N2—C7—C6	−173.9 (2)	C22—C23—C24—C19	1.9 (5)
C14—N2—C7—C6	52.3 (3)	C22—C23—C24—C25	−178.4 (3)
C5—C6—C7—C8	5.3 (4)	C20—C19—C24—C23	−1.4 (4)
F1—C6—C7—C8	−172.7 (2)	C18—C19—C24—C23	179.2 (3)
C5—C6—C7—N2	−177.2 (2)	C20—C19—C24—C25	178.9 (3)
F1—C6—C7—N2	4.8 (4)	C18—C19—C24—C25	−0.5 (5)
N2—C7—C8—C9	−179.8 (2)	C23—C24—C25—O7	−8.7 (5)
C6—C7—C8—C9	−2.4 (4)	C19—C24—C25—O7	171.0 (3)
C5—C4—C9—N3	−175.6 (2)	C23—C24—C25—O6	171.3 (4)
C3—C4—C9—N3	3.5 (4)	C19—C24—C25—O6	−9.0 (6)

Symmetry codes: (i) −*x*, −*y*, −*z*+1.

### Hydrogen-bond geometry (Å, °)

**Table:**

*D*—H···*A*	*D*—H	H···*A*	*D*···*A*	*D*—H···*A*
OW1—HW1B···O5	0.84 (3)	2.43 (3)	3.108 (4)	139 (4)
N1—H1A···O7^ii^	0.90	1.82	2.717 (3)	174.
N1—H1B···O1^iii^	0.90	1.79	2.688 (3)	173.
N1—H1B···O2^iii^	0.90	2.60	3.246 (3)	130.
OW1—HW1A···O3^i^	0.84 (3)	2.12 (1)	2.937 (3)	164 (4)
O6—H6···O5	0.85 (4)	1.53 (4)	2.379 (4)	175 (8)

Symmetry codes: (ii) *x*+1/2, −*y*+1/2, *z*+1/2; (iii) *x*+1, *y*, *z*+1; (i) −*x*, −*y*, −*z*+1.
